# Clinical Implications of Non-Steatotic Hepatic Fat Fractions on Quantitative Diffusion-Weighted Imaging of the Liver

**DOI:** 10.1371/journal.pone.0087926

**Published:** 2014-02-04

**Authors:** Hildebrand Dijkstra, Astri Handayani, Peter Kappert, Matthijs Oudkerk, Paul E. Sijens

**Affiliations:** 1 Center for Medical Imaging - North East Netherlands, University of Groningen, University Medical Center Groningen, Groningen, The Netherlands; 2 Department of Radiology, University of Groningen, University Medical Center Groningen, Groningen, The Netherlands; Oregon Health & Science University, United States of America

## Abstract

Diffusion-weighted imaging (DWI) is an important diagnostic tool in the assessment of focal liver lesions and diffuse liver diseases such as cirrhosis and fibrosis. Quantitative DWI parameters such as molecular diffusion, microperfusion and their fractions, are known to be affected when hepatic fat fractions (HFF) are higher than 5.5% (steatosis). However, less is known about the effect on DWI for HFF in the normal non-steatotic range below 5.5%, which can be found in a large part of the population. The aim of this study was therefore to evaluate the diagnostic implications of non-steatotic HFF on quantitative DWI parameters in eight liver segments. For this purpose, eleven healthy volunteers (2 men, mean-age 31.0) were prospectively examined with DWI and three series of in-/out-of-phase dual-echo spoiled gradient-recalled MRI sequences to obtain the HFF and T_2_*. DWI data were analyzed using the intravoxel incoherent motion (IVIM) model. Four circular regions (ø22.3 mm) were drawn in each of eight liver segments and averaged. Measurements were divided in group 1 (HFF≤2.75%), group 2 (2.75< HFF ≤5.5%) and group 3 (HFF>5.5%). DWI parameters and T_2_* were compared between the three groups and between the segments. It was observed that the molecular diffusion (0.85, 0.72 and 0.49 ×10^−3 ^mm^2^/s) and T_2_* (32.2, 27.2 and 21.0 ms) differed significantly between the three groups of increasing HFF (2.18, 3.50 and 19.91%). Microperfusion and its fraction remained similar for different HFF. Correlations with HFF were observed for the molecular diffusion (r = −0.514, p<0.001) and T_2_* (−0.714, p<0.001). Similar results were obtained for the majority of individual liver segments. It was concluded that fat significantly decreases molecular diffusion in the liver, also in absence of steatosis (HFF≤5.5%). Also, it was confirmed that fat influences T_2_*. Determination of HFF prior to quantitative DWI is therefore crucial.

## Introduction

The effect of fat on the self-diffusion of water has been assessed since the onset of nuclear magnetic resonance. Already in 1983, it was demonstrated that water diffusion drops six-fold inside of Cheddar and Swiss cheeses [1]. Later it was observed in vitro that water diffusion is hindered by lipid-rich cores in susceptible plaque [2]. The clinical assessment of water diffusion in the liver became feasible with the introduction of diffusion weighted imaging (DWI) in the abdomen [3]. DWI reflects the mobility of water molecules (molecular diffusion) in a tissue which can be described by the apparent diffusion coefficient (ADC) or the intravoxel incoherent motion (IVIM) model [4]–[6]. Since then DWI has been successfully applied in the assessment of focal liver lesions and diffuse liver diseases such as cirrhosis, fibrosis and steatosis [7]–[11]. However, the effect of fat on hepatic DWI is still subject of debate. In an animal study it was concluded that steatosis may confound determination of hepatic fibrosis with DWI [12]. This was confirmed in two clinical studies where the ADC decreased significantly in patients with hepatic steatosis [13], [14]. Similarly, a study which applied the IVIM model demonstrated that steatosis can reduce the molecular diffusion significantly and thus act as a potential confounder when IVIM is used to assess diffuse liver diseases such as cirrhosis [15].

These studies discussed the effect of hepatic fat on DWI for patients with steatosis, which is defined as fat fractions higher than 5.56% [16]. However, a detailed insight in the dependency of IVIM parameters on normal (non-steatotic) fat fractions ranging between 0 and 5.56% has not been reported yet; it has been suggested however that there might be a nonlinear relationship [15]. Also, the relation between the different segmental regions of the liver and the effect of fat on IVIM modelled DWI has not been studied up to now. Considering that fat content has been demonstrated to differ between liver segments, its effect on IVIM modelled DWI may be expected to be location dependent [17]. In addition, it has been reported that next to the effects of fat on diffusion, fat also affects T_2_* estimation [18]–[20]. The purpose of this study was therefore to evaluate the diagnostic implications of non-steatotic HFF (< 5.5%) on quantitative DWI by assessing the HFF and T_2_* of healthy subjects in eight liver segments.

## Materials and Methods

### Ethics statement

The protocol of the study was approved by the Medical Ethics Review Board of the University Medical Center Groningen, and written informed consent was obtained for each volunteer.

### Study population

In April 2011, healthy volunteers were randomly selected by local advertisement in the university to ensure a diverse population. Volunteers were required to be without any history of hepatic pathology or any other pathology related to liver function. The minimum age for inclusion was 18 years old. Exclusion criteria included MRI contra-indications such as pacemakers, clips, stents and implants. In total, 11 subjects were included (2 men) with an age between 18 to 56 years old (mean 31.0) and a body mass between 55 and 116 kg. Body-mass-index (BMI) ranged between 19.9 and 34.4 kg/m^2^ (mean 25.4 kg/m^2^). The only preparation before the examination was an 8-h fasting period.

### MR protocols

All subjects were prospectively examined on a 1.5 T MRI system (Magnetom Avanto, Siemens Medical Solutions, Erlangen, Germany). The body coil served as transmitter and a 24-element spine matrix coil in combination with a 6-element body matrix as receiver.

After the localiser scans, a series of diffusion weighted images (DWI) were obtained using a spin echo based single shot echo-planar imaging (SS-EPI) sequence in combination with spectral adiabatic inversion recovery (SPAIR) fat suppression. The DWI acquisitions (b =  0, 50, 100, 250, 500, 750 and 1000 s/mm^2^) were gated using PACE respiratory triggering (TR = 3065–5947 ms) and tuned with the following parameters: TE 90 ms; FA 90°; slice-thickness 5 mm; FOV 300×242 mm^2^; matrix 144×116; bandwidth 1335 Hz/pixel; 4 averages and parallel acquisition technique GRAPPA with acceleration factor 2. Diffusion gradients (25 mT/m) were applied in the phase-, read-, and z-directions separately using bipolar diffusion-encoding schemes. For each subject, 16 transverse slices were acquired in interleaved mode to cover the liver in an acquisition time between 7.2 and 13.5 minutes.

After the DWI scans, a dual-echo spoiled gradient recalled (SPGR) sequence was acquired to obtain two series of in-phase (IP) images with echo times of 4.5 and 18 ms, TR = 220 ms and FA = 70° to calculate T_2_*. Then, to calculate the hepatic fat fraction (HFF), a second dual-echo SPGR was acquired to obtain two series of both out-phase (OP) and in-phase (IP) images with echo times of 2.38 and 4.76 ms respectively tuned with TR = 206 ms and FA = 70°. Finally, a third series of dual-echo SPGR was acquired with equal TE/TR settings as the second series hence a flip angle of 20°. All three SPGR scans were acquired with slice-thickness 6 mm; FOV 375×196 mm^2^; matrix 256×134; bandwidth 434 Hz/pixel; 1 averages and parallel acquisition technique GRAPPA with acceleration factor 2 and an acquisition time between 1.5 and 2.0 minutes. Total acquisition time for DWI and HFF measurements was between 12 and 20 minutes.

### Fitting of DWI signal

Bi-exponential fitting procedures and exact positioning of ROIs were performed using a programmable graphical and calculus environment (Matlab, The Mathworks, Natick, MA, USA) according to the instructions of a radiologist (M.O.) with more than 35 years of experience. For all analyses, the diffusion weighted signal intensities S were fitted bi-exponentially using the parameters prescribed by the IVIM model [4], [21]: 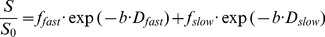
(1)


where S_0_ is the maximum signal intensity, D_fast_ is the fast pseudodiffusion component, f_fast_ is the fraction of the fast component, D_slow_ is the slow diffusion component and f_slow_ is the fraction of the slow component (f_slow_ = 1 – f_fast_) as defined previously by Le Bihan et al. [21]. In this study, D_fast_ is referred to as microperfusion, and f_fast_ as the fraction of microperfusion in accordance with the study of Lemke et al. who suggested that the IVIM-model separates DWI measurements into a “contribution of microperfusion and diffusion” [22]. D_slow_ is referred to as the molecular diffusion in accordance with the study of Luciani et al. [23].

Equation 1 was fitted by the Nelder-Mead simplex direct search method with bound constraints, which performs a constrained non-linear minimisation of the sum of the squared residuals [3], [24]. The initial guess D^0^_slow_ was estimated by calculating the slope of the asymptote of the slow signal component between b = 500 and 1000 s/mm^2^, and D_slow_ was bound between 0.5 and 5×D^0^_slow_×10^−3 ^mm^2^/s. The intercept of the asymptote with the y-axis at S_0_ resulted in an initial guess f^0^_fast_, and f_fast_ was bound between f^0^_fast_ – 0.02 and f^0^_fast_ + 0.02. The slope of the signal between b = 0 and b = 100 s/mm^2^ was used to guess the initial value of the fast signal component (D^0^_fast_), and D_fast_ was bound between D_slow_ and 100×10^−3 ^mm^2^/s.

### Hepatic fat fraction

The hepatic fat fraction (HFF) was calculated by Dixon’s in- and out-of-phase SPGR imaging modified with dual flip angles (70°, 20°) as proposed by Hussain et al.: HFF  =  HFF_20_ if HFF_20_ ≤ HFF_70_ and otherwise HFF  =  100 – HFF_20_ [25], [26]. The second SPGR series with a flip angle of 70 degrees were used to calculate HFF_70_: 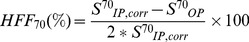
(2)


where
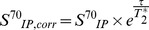
(3)


and S^70^_IP_ and S^70^_OP_ are the signal intensities of the IP and OP images of the second SPGR series using τ = 2.38 ms (TE_IP_ – TE_OP_). Similarly, the calculation of HFF_20_ was done using the third SPGR series with a flip angle of 20°. T_2_* was estimated using the first dual echo SPGR series:
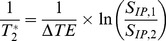
(4)


where ΔTE = 13.5 ms (TE_IP,2_-TE_IP,1_) and S_IP,1_ and S_IP,2_ are the respective signal intensities of both echoes.

### Image analysis

First the DWI data were loaded. For each of the 11 subjects, four circular regions-of-interest (ROI) with a diameter of 22.3 mm were drawn in each of the eight segmental regions (II – VIII) according to the Couinaud-Bismuth classification [27], [28]. The four ROIs were drawn on four different slices when possible; hence when no additional slices were available a second ROI was drawn on the same slice (yet in another location of the segment). For each ROI the average signal intensity S was obtained and the IVIM-DWI parameters (D_slow_, D_fast_ and the respective fractions) were fitted. The exact locations of the ROIs were stored as xy-coordinates, and for each ROI the HFF and T_2_* were recorded. Finally, the four ROIs measured in each segment were averaged, resulting in 88 measurements totally (11 subjects, 8 segments). During the assessments, any visible vascular and biliary structures nearby were avoided.

### Statistical analysis

Statistical analyses were performed using SPSS (SPSS 20, Chicago, IL, USA). All data were tested for normality using Shapiro–Wilk tests. Non-steatotic measurements (HFF ≤ 5.5%) were divided into two groups: group 1 (HFF ≤ 2.75%) and group 2 (2.75 < HFF ≤ 5.5%). Steatotic measurements (HFF > 5.5%) were assigned to group 3. For normally distributed data (D_slow_, D_fast_, f_fast_ and T_2_*) one-way ANOVA tests were used to compare measurements between the three groups. Post-hoc comparisons after ANOVA were implemented using Fisher’s LSD tests which provides familywise type I error protection when the number of comparisons equals three, while providing increased power compared to Bonferrorni correction [29]. For non-normally distributed data (HFF) the differences between the three groups were determined using non-parametric Kruskal-Wallis tests, where after post-hoc Mann-Whitney U multiple comparisons were performed with Bonferroni type I error correction.

Guiu et al. reported a potential nonlinear effect between D_slow_ and HFF, especially for HFF below 3% [15]. To investigate whether the relationship between HFF and D_slow_, D_fast_, f_fast_ and T_2_*, was linear or potentially nonlinear, Pearson’s correlations for all data points were calculated using a linear (Y = a•HFF + b) and a log-linear (Y = a·log(HFF) + b) model. The best-fit model, with the highest correlations for all data points was used to investigate the individual Pearson’s correlations in each of the eight individual liver segments. The significance of the multiple correlation analysis was adjusted by Bonferroni correction to avoid type I errors. Correlations (in absolute values) were classified as weak (r < 0.36), moderate (r = 0.36 to 0.67) and strong (r > 0.67) [30]. For all statistical tests P < 0.05 was considered to indicate a statistically significant difference.

## Results

### Effect of fat on IVIM-DWI parameters

**Figure 1:**
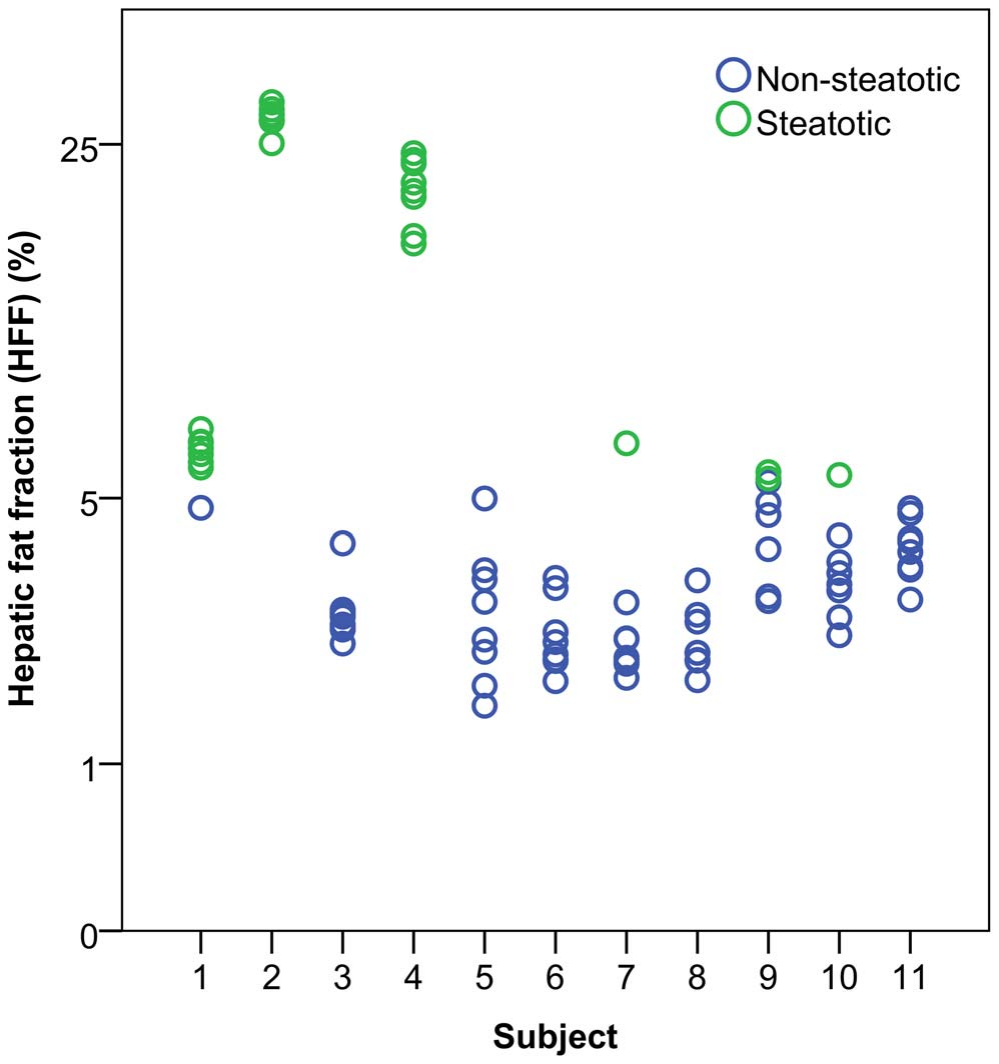
Distribution of HFF measurements in all subjects. For each subject (n = 11), four circular regions-of-interest (ø22.3 mm) were drawn in each of the eight segmental regions (II – VIII) according to the Couinaud-Bismuth classification and averaged, resulting in a total of 88 measurement points. Five subjects demonstrated non-steatotic HFF (≤5.5%) measurements only, two subjects had steatotic HFF (>5.5%) measurements only, and four subjects had both steatotic and non-steatotic measurements.

The HFF were non-normally distributed (p<0.001) and ranged between 1.5 and 29.9% for the eleven subjects. Five subjects yielded non-steatotic HFF (≤5.5%) measurements only, two subjects had steatotic HFF (>5.5%) measurements only, and four subjects had both steatotic and non-steatotic measurements (Fig. 1).

**Figure 2:**
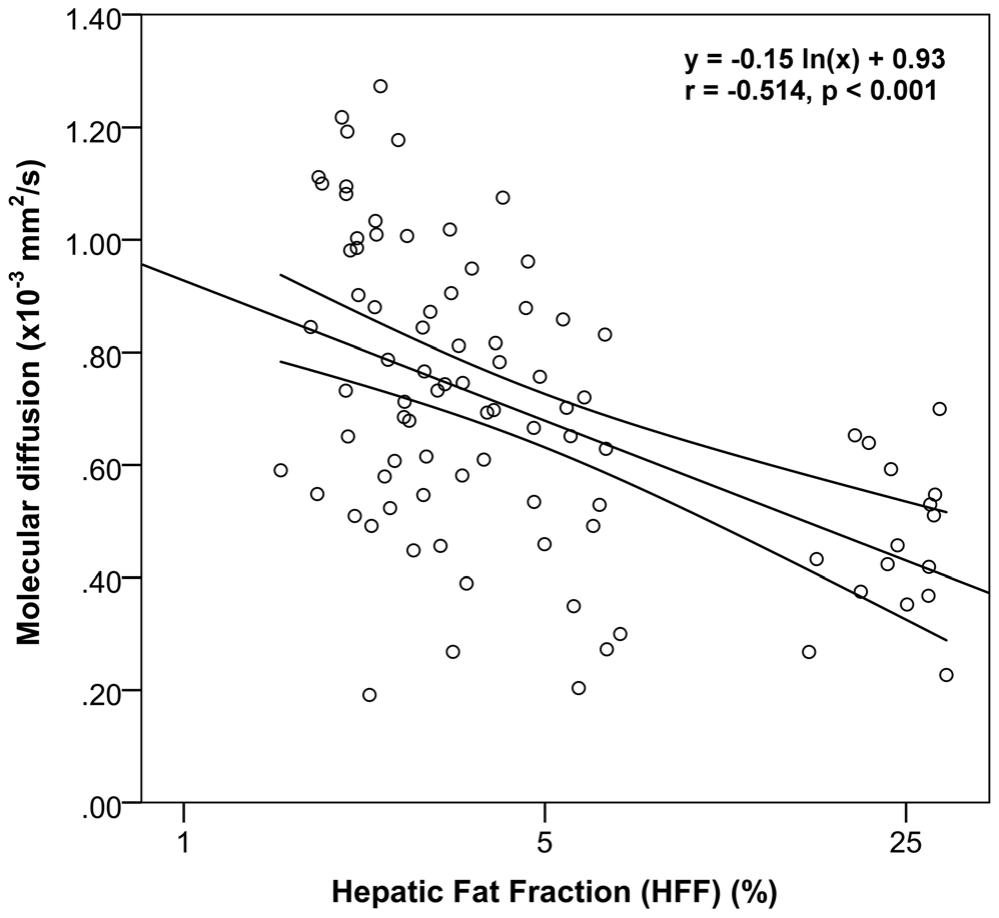
Regression plot between HFF and molecular diffusion. The correlation between HFF (%) and molecular diffusion (×10^−3 ^mm^2^/s) was assessed by using a log-linear model. Pearson’s product-moment correlation and its significance were calculated. The log-linear regression line is displayed together with its 95% confidence interval.

IVIM-DWI parameters were normally distributed (p≥0.319). Molecular diffusion (D_slow_) differed significantly between the three groups of different HFF, and also the intergroup comparisons were significantly different (p<0.001, Table 1). D_slow_ was 0.85×10^−3 ^mm^2^/s for the first group (HFF ≤ 2.75%) and decreased steadily to 0.72×10^−3 ^mm^2^/s in group 2 (2.75 < HFF ≤ 5.5%) and 0.49×10^−3 ^mm^2^/s in group 3 (HFF > 5.5%). D_fast_ and f_fast_ did not show differences between the three HFF groups (p≥0.194). Pearson’s correlation analysis (Table 2) showed a significant negative linear relationship with moderate correlation between HFF and D_slow_ using both the linear model (r = −0.446, p<0.001) and the log-linear model (r = −0.514, p<0.001, Fig. 2). The log-linear model showed overall higher correlations compared to the linear model and was therefore used for the individual segment analysis. The average HFF varied from 3.00±9.17% to 7.32±9.36% between the individual segments (Table 3). In segment VII a significant negative linear relationship with strong correlation (r = −0.840; p ≤ 0.008) was observed between HFF and D_slow_ using the log-linear model (Table 4). No significant correlations between HFF and D_fast_ or f_fast_ were observed.

**Table 1:** Comparison of hepatic fat fraction (HFF), IVIM-DWI parameters and T_2_* between groups.

	Non-steatotic		Steatotic				
	Group 1 HFF ≤ 2.75%	Group 2 2.75 < HFF ≤5.5%	Group 3 HFF > 5.5%	Intergroup test	Post-hoc multiple comparisons between groups		
	n = 31	n = 30	n = 27		1–2	1–3	2–3
HFF (%) ^†^	2.18±0.30	3.50±0.74	19.91±9.69	<0.001*	<0.001*	<0.001*	<0.001*
D_slow_ (10^−3 ^mm^2^/s) ^‡^	0.85±0.27	0.72±0.20	0.49±0.17	<0.001*	0.023*	<0.001*	<0.001*
D_fast_ (10^−3 ^mm^2^/s) ^‡^	37.9±8.35	39.6±8.86	42.4±10.5	0.194	0.477	0.072	0.267
f_fast_ (%) ^‡^	36±8.5	37±8.3	39±9.0	0.279	0.601	0.116	0.288
T_2_* (ms) ^‡^	32.2±5.1	27.2±3.4	21.0±3.7	<0.001*	<0.001*	<0.001*	<0.001*

Measurements acquired in the liver. **^†^** Data are medians ± median deviations and intergroup differences were assessed by Kruskal-Wallis tests and post-hoc Mann-Whitney U multiple comparisons with Bonferroni type I error correction. **^‡^** Data are means ± standard deviations and intergroup differences were assessed by one-way ANOVA and post-hoc multiple comparisons using Fisher’s LSD tests providing type I error protection as the number of comparisons equals 3. *P-value indicates significant difference.

**Table 2:** Correlations of IVIM-DWI parameters and T_2_* with hepatic fat fraction (HFF) using two models.

	Linear model			Log-linear model		
	Pearson’s r	F	P-value	Pearson’s r	F	P-value
**D_slow_**	−0.446*	21.317	<0.001*	−0.514*	30.926	<0.001*
**D_fast_**	+0.040	0.138	0.711	+0.101	0.892	0.348
**f_fast_**	−0.041	0.148	0.701	+0.030	0.075	0.785
T_2_*	−0.607*	50.277	<0.001*	−0.714*	89.190	<0.001*

Two models were used to assess the effect of hepatic fat fraction (HFF) on the IVIM-DWI parameters (D_slow_, D_fast_ and f_fast_) or T_2_* measured in 11 patients and 8 segments (n = 88). The linear model assumed a linear relationship between HFF and the IVIM-DWI parameters or T_2_* (Y = a•HFF+b). The log-linear model assumed a linear relationship between the logarithmic of HFF and the IVIM-DWI parameters or T_2_* (Y = a•log(HFF)+b). Pearson’s correlations increased when the log-linear model was applied. *Indicates significant correlations.

**Table 3:** Hepatic fat fraction (HFF), IVIM-DWI parameters and T_2_* per segment.

Seg	HFF ^†^ (%)	D_slow_^‡^ (10^−3 ^mm^2^/s)	D_fast_^‡^ (10^−3 ^mm^2^/s)	f_fast_^‡^ (%)	T_2_* ^‡^ (ms)
II	4.99±8.80	0.48±0.28	48.4±8.5	50±5.1	25.5±5.8
III	7.32±9.36	0.65±0.16	40.9±8.8	44±6.7	28.1±5.9
IVa	3.29±8.64	0.85±0.28	40.0±7.0	38±5.4	26.8±8.4
IVb	3.00±9.17	0.85±0.19	48.4±8.6	39±6.1	29.0±7.1
V	3.46±7.38	0.60±0.25	38.0±8.1	33±5.9	27.6±4.8
VI	3.41±8.73	0.74±0.21	37.5±7.2	32±5.3	27.7±5.8
VII	3.98±9.18	0.75±0.23	34.3±6.6	31±5.6	26.2±6.0
VIII	3.27±8.54	0.59±0.26	31.4±5.8	29±5.3	25.6±5.6

For each subject four ROIs were drawn in each segment and then averaged, resulting in 11 measurements per segment. **^†^** Data are medians ± median. **^‡^** Data are means ± standard deviations.

**Table 4:** Correlations with hepatic fat fraction (HFF) per segment.

Seg	D_slow_		D_fast_		f_fast_		T_2_*	
	r	p	r	p	r	p	r	p
II	−0.328	1.000	0.497	0.960	−0.213	1.000	−0.747	0.064
III	−0.144	1.000	0.434	1.000	−0.182	1.000	−0.406	1.000
IVa	−0.624	0.320	0.429	1.000	0.417	1.000	−0.767*	0.048*
IVb	−0.528	0.760	0.260	1.000	0.277	1.000	−0.801*	0.024*
V	−0.732	0.080	−0.345	1.000	−0.091	1.000	−0.692	0.144
VI	−0.669	0.192	−0.241	1.000	−0.043	1.000	−0.767*	0.048*
VII	−0.840*	0.008*	0.020	1.000	0.074	1.000	−0.777*	0.040*
VIII	−0.639	0.304	−0.177	1.000	−0.026	1.000	−0.804*	0.024*

Pearson’s r correlation coefficients were calculated per segment between the HFF and the IVIM-DWI parameters (D_slow_, D_fast_ and f_fast_) or T_2_* using the log-linear model. *Indicates a significant correlation (adjusted for type I errors using Bonferroni correction).

### Effect of fat on T_2_*

**Figure 3:**
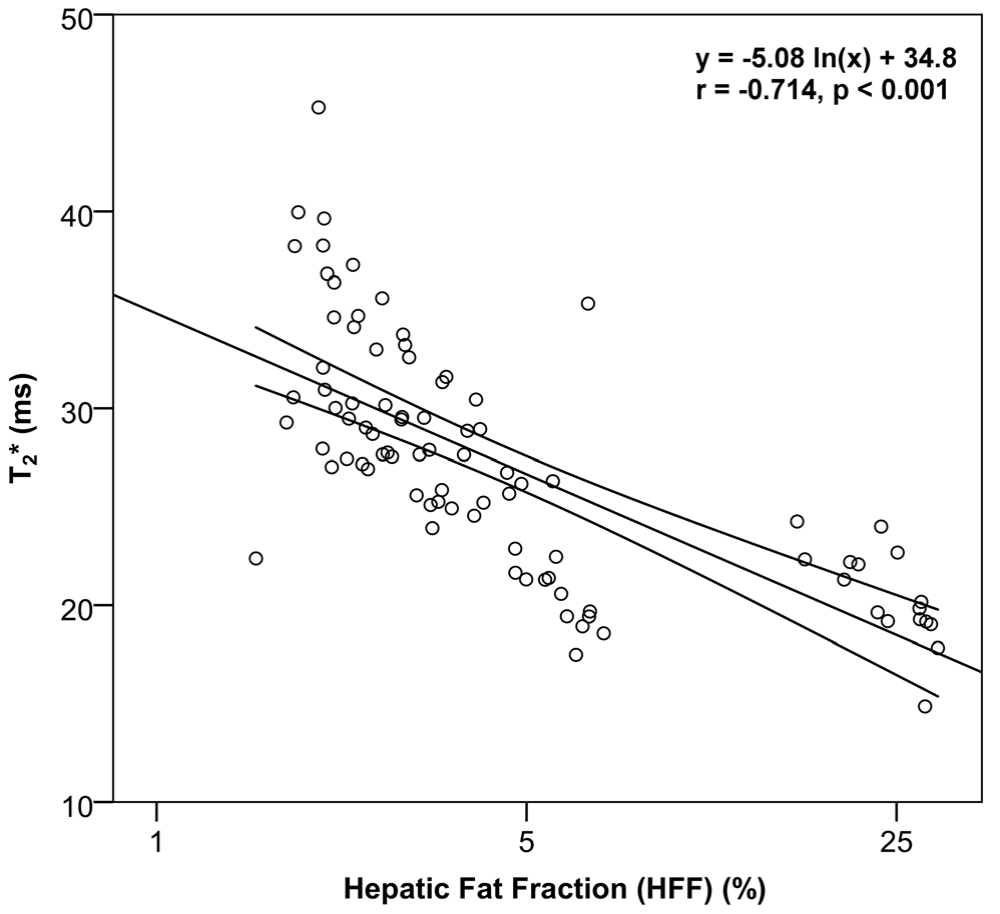
Regression plot between HFF and T_2_*. The correlation between HFF (%) and T_2_* (ms) was assessed by using a log-linear model. Pearson’s product-moment correlation and its significance were calculated. The log-linear regression line is displayed together with its 95% confidence interval.

T_2_* was normally distributed (p = 0.116) and differed significantly between the three groups of different HFF, and also the intergroup comparisons were significantly different (p<0.001, Table 1). T_2_* was 32.2 ms for the first group (HFF ≤ 2.75%) and decreased steadily to 27.2 ms in group 2 (2.75 < HFF ≤ 5.5%) and 21.0 in group 3 (HFF > 5.5%). Pearson’s correlation analysis (Table 2) showed a significant negative linear relationship with moderate to strong correlation between HFF and T_2_* using both the linear model (r = −0.607, p<0.001) and the log-linear model (r = −0.714, p<0.001, Fig. 3). The log-linear model showed overall higher correlations compared to the linear model and was therefore used for the individual segment analysis. In 5 of 8 segments a significant negative linear relationship with strong correlation (r = −0.767 to −0.804; p ≤ 0.048) was observed between HFF and T_2_* using the log-linear model (Table 4).

## Discussion

### Effect of fat on IVIM-DWI parameters

In this study it was demonstrated that molecular diffusion (D_slow_) in the liver is affected by hepatic fat, also in the absence of steatosis (HFF below 5.5%). D_slow_ differed significantly between three groups of different HFF, and a steady significant decrease of D_slow_ with moderate correlation was found for increasing HFF. These results complement existing knowledge of the reduction of molecular diffusion by steatotic HFF (>5.5%). Previous IVIM studies showed comparable negative correlations (r = −0.59 and r = −0.18) between HFF and molecular diffusion using a linear model [15], [31]. In addition, Guiu et al. noticed a potential nonlinear effect between D_slow_ and HFF, especially for HFF below 3% [15]. This was confirmed in our study: the relationship between HFF and D_slow_ appeared nonlinear with higher correlations for the log-linear model compared to the linear model. Others compared non-steatotic with steatotic livers and observed a significant decrease of D_slow_ from 1.24×10^−3 ^mm^2^/s to 1.03×10^−3 ^mm^2^/s [15]. Mono-exponential fitting of just a pair of DW images also indicated a significant decrease of the ADC in patients with HFF higher than 5%, with moderate correlations (r = −0.39) [13]. In an animal study similar results were found, increasing degrees of hepatic steatosis correlated fairly well (r = −0.56) with decreasing liver ADCs [12].

With regard to the effect of fat on IVIM-DWI parameters in the different segmental regions, we used the log-linear model which had the highest correlations in the overall analysis compared to the linear model. It was observed that D_slow_ correlated moderately to strong with HFF; however only in segment VII the correlation reached significance. This indicates that fat has a rather similar effect on D_slow_ throughout the liver, yet larger studies are needed to confirm these findings. In particular the segments in the left lobe (II and III) did not demonstrate a relationship between D_slow_ and HFF, which may reflect increased cardiac motion artifacts in that region, hampering DWI quantification [32].

**Figure 4:**
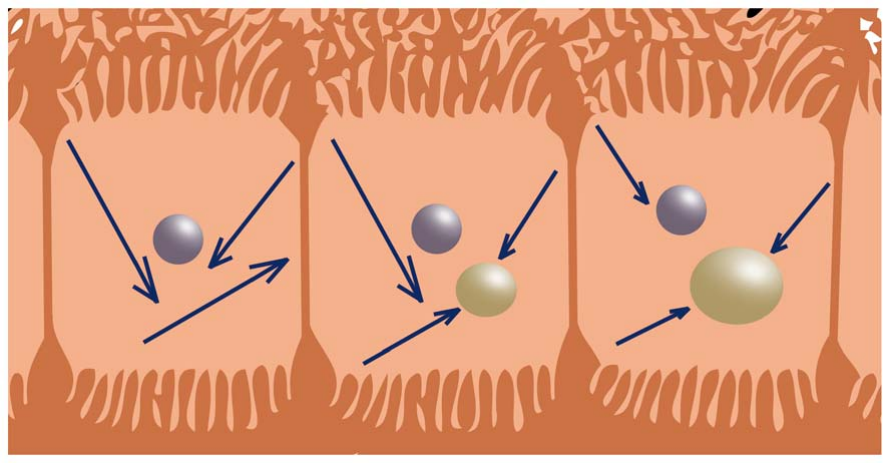
Schematic representation of the reduction of molecular diffusion in the hepatocytes by fat droplets. When fat is present in the liver, it is stored as triglycerides in sphere shaped vacuoles. Commonly, these (macrovesicular) vacuoles appear as just one large droplet (yellow spheres) with a diameter larger than 15 µm, sometimes dislocating the nucleus (purple spheres) with it. The mean diameter of a hepatocyte ranges between 20 and 40 µm. A considerable fraction of the volume of the hepatocyte can therefore be occupied by the macrovesicular fat droplet. Considering a diffusion length of about 17 µm, which is in the same order of magnitude of the hepatocyte’s diameter, the movement of water molecules (blue arrows) can be physically hindered by the presence of macrovesicular fat droplets in hepatocytes. This explains the decrease of the molecular diffusion with increasing hepatic fat fractions as a mechanical process.

The decrease of molecular diffusion by fat can be due to several mechanisms. MR relaxation is determined predominantly by water-macromolecular interactions [33]. The MR signal from protons bound to macromolecules such as fat will interfere with the MR signal from freely diffusing water molecules. This can partly explain the observed decreased molecular diffusion in the presence of fat. To prevent interference, fat suppression techniques serve to suppress the signal originating from protons bound to fat in order to reduce the chemical shift artifacts and eliminate signals arising from adipose tissue [34]. However, fat suppression techniques are not perfect and come with disadvantages and pitfalls such as the dependency on the homogeneity of the main static magnetic field. Hence there will always be some interference of MR signal between free protons and macromolecular protons that cannot be neglected. However, as indicated previously by the breast DWI study of Baron et al., low molecular diffusion in the presence of fat may reflect either direct contributions from the protons of the relatively immobile fat molecules or low water content, thereby restricting the diffusion of water (trapped water) [35]. Similarly, we hypothesize that the reduction of molecular diffusion in the liver is caused by physical hindrance of the movement of water molecules by the presence of macrovesicular fat droplets in hepatocytes. The fat present in the liver, is stored as triglycerides in sphere shaped vacuoles, which usually appear as large droplets with diameters larger than 15 µm [36]. These vacuoles reside in the hepatocytes, which are polygonal cells with six or more faces and a mean diameter ranging between 20 and 40 µm [37]. A considerable fraction of the volume of the hepatocyte can therefore be occupied by the macrovesicular fat droplet (Fig. 4). Considering a diffusion length [38] of about 17 µm (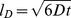, D = 1.0×10^−3 ^mm^2^/s, t =  50 ms), which is in the same order of magnitude of the hepatocyte’s diameter, we suspect that the movement of water molecules can be physically hindered by the presence of macrovesicular fat droplets in hepatocytes. This would be a mechanical process rather than signal interference between protons bound to fat molecules and free water protons.

The microperfusion parameters (D_fast_ and f_fast_) were overall not affected by the HFF, neither in the individual segments. The average fraction of microperfusion f_fast_ was comparable to previously published numbers (29–35%) on healthy livers [15], [23]. In agreement with earlier findings, f_fast_ was highest (≥44%) in the left lobe (segments II and III) [39]. Microperfusion (D_fast_) has been found to be lower in patients with steatosis compared to patients without steatosis [15]. In contrast, in this study we did not find a relation between D_fast_ and HFF. This can be partially due to the limited accuracy of D_fast_ in this study. Because of software limitations on the MR system, it was not possible to acquire any data of D_fast_ between b = 0 and 50 s/mm^2^. It is known that the choice of b-values is important for an accurate determination of IVIM parameters, and especially for a precise estimate of D_fast_ a number of b-values should be in the range from b = 0 to 50 s/mm^2^ [22]. The lack of b-values below 50 s/mm^2^ can also explain the relatively low standard deviation of D_fast_ in our study. Previously, the ratio of D_fast_ and its standard deviation has been reported to range roughly between 1 and 3, compared to 4 – 4.5 in our study [15], [23]. This suggests that in our study D_fast_ is potentially biased and forced by the fitting algorithm towards a relatively fixed value due to a lack of underlying data points, thereby reducing the overall standard deviation.

The wide range of HFF measurements (1.5−29.9%) demonstrated by our subjects was in concordance with previously published numbers. In a large population-based project conducted in northeast Germany, HFF ranged between 4.6% and 34.9% for the majority of a group of 88 healthy volunteers [40]. Also in a comparative methodological study, HFF of healthy volunteers ranged up to 21.1% showing high correlations between MR spectroscopy and two-point Dixon-based MRI fat quantification [41].

### Effect of fat on T_2_*

In this study, T_2_* differed significantly between three groups of different HFF, also for non-steatotic HFF below 5.5%, and a steady significant decrease of T_2_* with strong correlation was found for increasing HFF. Also in the different segmental regions, we found that T_2_* correlated significantly with HFF in 5 of the 8 segments. Correlations were higher for the log-linear model compared to the linear model, suggesting a nonlinear relationship between HFF and T_2_* as well. Hepatic T_2_* variations among different segments have been shown to be low in healthy subjects, ranging between 19.3 and 29.9 ms [42], which is in accordance with our observations. Similar results were obtained in an animal study where the T_2_* of liver parenchyma of rats decreased from 31.4 ms for the control group (0.9% HFF) to 19.1 ms for rats fed by a four week choline-deficient diet (26.0% HFF) [43]. A clinical study on patients with non-alcoholic fatty liver disease (NAFLD) reported significant decreases of T_2_ relaxation times of water with increasing fat fractions [19]. In addition, T_2_* shorting by fat has been confirmed using various phantoms with different fat-water mixtures [44].

However, for a number of studies no correlation was found between T_2_* relaxation and HFF [20], [45]. For example, Hernando and Kühn et al. demonstrated that T_2_* estimations are inaccurate in tissues with high fat content due to the complex fat spectrum, and concluded that these issues can be solved when multipeak spectral modeling of fat is applied: this way they showed that T_2_* is independent of the fat fraction [18], [40]. In our study, T_2_* was not corrected for the spectral complexity of the fat signal, which can explain the dependency of T_2_* on the HFF in our study.

The dependency of T_2_* on HFF can also be understood from the perspective of Bottomley et al. who suggested a fast exchange two-state (FETS) model to describe proton T_1_ and T_2_ relaxation in normal tissue [46]. They indentified three chemically different proton species: macromolecular protons (excluding fatty acids), free water protons, and mobile fatty acid protons, relaxing with T_2_ times of ∼10–100 µs, ∼50 ms and ∼0.2 s respectively. If the amount of fat in the liver changes, the interference pattern of the different proton signals, causing dephasing, also changes, and the overall effect can be shortening of the T_2_* relaxation time. This is in concordance with Yu et al. who suggested that when fat coexists with water in a voxel, T_2_* relaxometry may be disturbed by the chemical shift of fat, due to constructive and destructive interference of fat and water signals [47].

### Clinical implications

It is known that the decreases of the molecular diffusion between normal liver tissue and cirrhosis or the different stages of fibrosis are relatively small and technically challenging to detect [23], [48], [49]. It is therefore important to know what methodological factors can reduce molecular diffusion, regardless of the pathology itself. One of these factors is that diffusion measurements can be heavily dependent on the MR-equipment used, which requires use of the same scanner to ensure comparable measurements [50]. Also, user-dependent factors such as the choice of measurement location within the liver may affect the diffusion measurements. This was demonstrated in a recent study where the apparent diffusion coefficient significantly depended on the segmental region in the liver [39]. In the current study, we added another factor: molecular diffusion is negatively related to the hepatic fat fraction, also at non-steatotic fat levels. This is especially important when in pursuit for quantitative cut-off values for molecular diffusion in order to discriminate healthy liver tissue from pathology. Molecular diffusion is dependent on the hepatic fat fraction, also below 5.5%. This implicates that any derived cut-off value of the molecular diffusion for cirrhosis, or stages of fibrosis, is dependent on the hepatic fat fraction as well, especially because it is known that hepatic fat fractions vary between subjects [51]. Therefore, we recommend that to correctly interpret quantitative hepatic DWI, acquisition of the hepatic fat fraction prior to the hepatic DWI protocol is necessary. In that way, diffusion measurements can be judged along with the fat measurement, which ensures a more reliable assessment of the diffusion properties of pathology.

Similar issues apply to quantitative evaluation of hemochromatosis using T_2_* (ms) estimation. The histopathologic iron grade can be classified using T_2_* measurement of the liver [52]. For that purpose cut-off values have been defined. However, if T_2_* values are dependent on the hepatic fat fraction, then cut-off values used in the classification of hemochromatosis ought to be corrected for the hepatic fat fraction.

Next to quantitative difficulties, also non-quantitative issues may arise due to the effect of fat on hepatic DWI. Hypointensity in DWI can be a side effect of T_2_* shortening. DW images are T_2_ weighted and changes in T_2_ relaxation times can therefore affect the signal intensities independently of the tissue diffusion in three ways [53]: shine-through (prolongation of T_2_), washout (balance between T_2_ prolongation and increased diffusion) and blackout or hypointensity (shortening of T_2_). As fat shortens T_2_*, locally elevated levels of fat in the liver can reveal as hypointense areas, which might be incorrectly interpreted as increased liver diffusion.

In conclusion, we have demonstrated that hepatic fat fractions significantly decrease the molecular diffusion in the liver, also for non-steatotic fat levels (≤5.5%). In addition, it was confirmed that hepatic fat fractions significantly decrease T_2_* measurements when multipeak spectral modeling is not applied. It is known that the decreases of the molecular diffusion between normal liver tissue and cirrhosis or the different stages of fibrosis are relatively small and technically challenging to be detected [23], [48], [49]. The knowledge of the effect of low levels of hepatic fat on the molecular diffusion is therefore expected to be of importance in the diagnosis and staging of fibrosis and cirrhosis using quantitative DWI. Therefore, we conclude that to correctly interpret quantitative hepatic DWI, acquisition of the hepatic fat fraction prior to the hepatic DWI protocol is necessary to ensure a reliable assessment of the diffusion properties of pathology.

## References

[1] Callaghan PT, Jolley KW, Humphrey RS (1983) Diffusion of fat and water in cheese as studied by pulsed field gradient nuclear magnetic resonance. J Colloid Interface Sci 93: 521–529.

[2] Toussaint J, Southern JF, Fuster V, Kantor HL (1997) Water diffusion properties of human atherosclerosis and thrombosis measured by pulse field gradient nuclear magnetic resonance. . Arterioscler Thromb Vasc Biol. 17: 542–546. 9102174 10.1161/01.atv.17.3.542

[3] Muller MF, Prasad P, Siewert B, Nissenbaum MA, Raptopoulos V, et al (1994) Abdominal diffusion mapping with use of a whole-body echo-planar system Radiology. 190: 475–478. 10.1148/radiology.190.2.8284402 8284402

[4] Le Bihan D, Breton E, Lallemand D, Aubin ML, Vignaud J, et al (1988) Separation of diffusion and perfusion in intravoxel incoherent motion MR imaging Radiology. 168: 497–505. 10.1148/radiology.168.2.3393671 3393671

[5] Yamada I, Aung W, Himeno Y, Nakagawa T, Shibuya H (1999) Diffusion coefficients in abdominal organs and hepatic lesions: Evaluation with intravoxel incoherent motion echo-planar MR imaging Radiology. 210: 617–623. 10.1148/radiology.210.3.r99fe17617 10207458

[6] Tachibana Y, Aida N, Niwa T, Nozawa K, Kusagiri K, et al (2013) Analysis of multiple B-value diffusion-weighted imaging in pediatric acute encephalopathy. PLoS One 8: e63869. 23755112 10.1371/journal.pone.0063869 PMC3670889

[7] Ichikawa T, Haradome H, Hachiya J, Nitatori T, Araki T (1998) Diffusion-weighted MR imaging with a single-shot echoplanar sequence: Detection and characterization of focal hepatic lesions. AJR Am J Roentgenol 170: 397–402. 9456953 10.2214/ajr.170.2.9456953

[8] Amano Y, Kumazaki T, Ishihara M (1998) Single-shot diffusion-weighted echo-planar imaging of normal and cirrhotic livers using a phased-array multicoil. Acta Radiol 39: 440–442. 9685834 10.1080/02841859809172460

[9] Kele PG, van der Jagt EJ (2010) Diffusion weighted imaging in the liver World J Gastroenterol. 16: 1567–1576. 10.3748/wjg.v16.i13.1567 PMC2848365 20355235

[10] Bakan AA, Inci E, Bakan S, Gokturk S, Cimilli T (2012) Utility of diffusion-weighted imaging in the evaluation of liver fibrosis. Eur Radiol 22: 682–687. 21984447 10.1007/s00330-011-2295-z

[11] Morelli JN, Michaely HJ, Meyer MM, Rustemeyer T, Schoenberg SO, et al (2013) Comparison of dynamic and liver-specific gadoxetic acid contrast-enhanced MRI versus apparent diffusion coefficients. PLoS One 8: e61898. 23805174 10.1371/journal.pone.0061898 PMC3689764

[12] Anderson SW, Soto JA, Milch HN, Ozonoff A, O'Brien M, et al (2011) Effect of disease progression on liver apparent diffusion coefficient values in a murine model of NASH at 11.7 tesla MRI. J Magn Reson Imaging 33: 882–888. 21448953 10.1002/jmri.22481

[13] Poyraz AK, Onur MR, Kocakoc E, Ogur E (2012) Diffusion-weighted MRI of fatty liver. J Magn Reson Imaging 35: 1108–1111. 22170763 10.1002/jmri.23519

[14] Wignall O, Scurr E, Collins D, Thng CH, Koh DM. (2008) Hepatic steatosis results in a reduction in the apparent diffusion coefficient (ADC) of liver parenchyma. Proc. Intl. Soc. Mag. Reson. Med. 16.

[15] Guiu B, Petit JM, Capitan V, Aho S, Masson D, et al (2012) Intravoxel incoherent motion diffusion-weighted imaging in nonalcoholic fatty liver disease: A 3.0-T MR study. Radiology 265: 96–103. 22843768 10.1148/radiol.12112478

[16] Szczepaniak LS, Nurenberg P, Leonard D, Browning JD, Reingold JS, et al (2005) Magnetic resonance spectroscopy to measure hepatic triglyceride content: Prevalence of hepatic steatosis in the general population. Am J Physiol Endocrinol Metab 288: E462–8. 15339742 10.1152/ajpendo.00064.2004

[17] Capitan V, Petit JM, Aho S, Lefevre PH, Favelier S, et al (2012) Macroscopic heterogeneity of liver fat: An MR-based study in type-2 diabetic patients. Eur Radiol 22: 2161–2168. 22562090 10.1007/s00330-012-2468-4

[18] Kuhn JP, Hernando D, Munoz Del Rio A, Evert M, Kannengiesser S, et al (2012) Effect of multipeak spectral modeling of fat for liver iron and fat quantification: Correlation of biopsy with MR imaging results. Radiology 265: 133–142. 22923718 10.1148/radiol.12112520 PMC3447175

[19] Gilman AJ, Qayyum A, Nystrom M, Noworolski SM. (2011) Liver fat and water MR T2 values at 3T: Dependence upon steatosis level. Proc. Intl. Soc. Mag. Reson. Med. 19.

[20] Thomsen C, Becker U, Winkler K, Christoffersen P, Jensen M, et al (1994) Quantification of liver fat using magnetic resonance spectroscopy. Magn Reson Imaging 12: 487–495. 8007779 10.1016/0730-725x(94)92543-7

[21] Le Bihan D, Turner R, Moonen CT, Pekar J (1991) Imaging of diffusion and microcirculation with gradient sensitization: Design, strategy, and significance J Magn Reson Imaging. 1: 7–28. 10.1002/jmri.1880010103 1802133

[22] Lemke A, Laun FB, Simon D, Stieltjes B, Schad LR (2010) An in vivo verification of the intravoxel incoherent motion effect in diffusion-weighted imaging of the abdomen. Magn Reson Med 64: 1580–1585. 20665824 10.1002/mrm.22565

[23] Luciani A, Vignaud A, Cavet M, Nhieu JT, Mallat A, et al (2008) Liver cirrhosis: Intravoxel incoherent motion MR imaging--pilot study Radiology. 249: 891–899. 10.1148/radiol.2493080080 19011186

[24] Turner R, Le Bihan D, Maier J, Vavrek R, Hedges LK, et al (1990) Echo-planar imaging of intravoxel incoherent motion Radiology. 177: 407–414. 10.1148/radiology.177.2.2217777 2217777

[25] Hussain HK, Chenevert TL, Londy FJ, Gulani V, Swanson SD, et al (2005) Hepatic fat fraction: MR imaging for quantitative measurement and display--early experience. Radiology 237: 1048–1055. 16237138 10.1148/radiol.2373041639

[26] Dixon WT (1984) Simple proton spectroscopic imaging. Radiology 153: 189–194. 6089263 10.1148/radiology.153.1.6089263

[27] Couinaud C. (1957) Le foie: Etudes anatomiques et chirurgicales. Paris: Masson.

[28] Bismuth H (1982) Surgical anatomy and anatomical surgery of the liver. World J Surg 6: 3–9. 7090393 10.1007/BF01656368

[29] Levin JR, Serlin RC, Seaman MA (1994) A controlled, powerful multiple-comparison strategy for several situations. Psychol Bull 115: 153–159.

[30] Taylor R (1990) Interpretation of the correlation-coefficient - a basic review. Journal of Diagnostic Medical Sonography 6: 35–39.

[31] Yu S, Kim S, Paek M, Goo E, Ji Y, et al. (2013) Correlation between hepatic fat content using 3-echo 3-D dixon method and intravoxel incoherent motion (IVIM) perfusion MR imaging. Applied Magnetic Resonance : 1–11.

[32] Kwee TC, Takahara T, Niwa T, Ivancevic MK, Herigault G, et al (2009) Influence of cardiac motion on diffusion-weighted magnetic resonance imaging of the liver. MAGMA 22: 319–325. 19727877 10.1007/s10334-009-0183-1

[33] Chai JW, Lin YC, Chen JH, Wu CC, Lee CP, et al (2001) In vivo magnetic resonance (MR) study of fatty liver: Importance of intracellular ultrastructural alteration for MR tissue parameters change. J Magn Reson Imaging 14: 35–41. 11436212 10.1002/jmri.1148

[34] Delfaut EM, Beltran J, Johnson G, Rousseau J, Marchandise X, et al (1999) Fat suppression in MR imaging: Techniques and pitfalls. Radiographics 19: 373–382. 10194785 10.1148/radiographics.19.2.g99mr03373

[35] Baron P, Dorrius MD, Kappert P, Oudkerk M, Sijens PE (2010) Diffusion-weighted imaging of normal fibroglandular breast tissue: Influence of microperfusion and fat suppression technique on the apparent diffusion coefficient. NMR Biomed 23: 399–405. 20131313 10.1002/nbm.1475

[36] Zaitoun AM, Al Mardini H, Awad S, Ukabam S, Makadisi S, et al (2001) Quantitative assessment of fibrosis and steatosis in liver biopsies from patients with chronic hepatitis C. . J Clin Pathol 54: 461–465. 11376020 10.1136/jcp.54.6.461 PMC1731453

[37] Kuntz E, Kuntz H, SpringerLink. (2008) Hepatology textbook and atlas.

[38] Chawla S, Kim S, Wang S, Poptani H (2009) Diffusion-weighted imaging in head and neck cancers. Future Oncol 5: 959–975. 19792966 10.2217/fon.09.77 PMC2791671

[39] Dijkstra H, Baron P, Kappert P, Oudkerk M, Sijens PE (2012) Effects of microperfusion in hepatic diffusion weighted imaging. Eur Radiol 22: 891–899. 22080250 10.1007/s00330-011-2313-1 PMC3297749

[40] Hernando D, Kuhn JP, Mensel B, Volzke H, Puls R, et al (2013) R2* estimation using "in-phase" echoes in the presence of fat: The effects of complex spectrum of fat. J Magn Reson Imaging 37: 717–726. 23055408 10.1002/jmri.23851 PMC3578028

[41] Irwan R, Edens MA, Sijens PE (2008) Assessment of the variations in fat content in normal liver using a fast MR imaging method in comparison with results obtained by spectroscopic imaging. Eur Radiol 18: 806–813. 17999066 10.1007/s00330-007-0801-0

[42] Meloni A, Luciani A, Positano V, De Marchi D, Valeri G, et al (2011) Single region of interest versus multislice T2* MRI approach for the quantification of hepatic iron overload. J Magn Reson Imaging 33: 348–355. 21274976 10.1002/jmri.22417

[43] Okada M, Katsube T, Kumano S, Kagawa Y, Araki T, et al (2011) Unenhanced fat fraction ratios obtained by MR and enhanced T2* values with liver-specific MR contrast agents for diagnosis of non-alcoholic steatohepatitis in rats. Acta Radiol 52: 658–664. 21498305 10.1258/ar.2011.100360

[44] Kamman RL, Bakker CJ, van Dijk P, Stomp GP, Heiner AP, et al (1987) Multi-exponential relaxation analysis with MR imaging and NMR spectroscopy using fat-water systems. Magn Reson Imaging 5: 381–392. 3695824 10.1016/0730-725x(87)90127-5

[45] Meisamy S, Hines CD, Hamilton G, Sirlin CB, McKenzie CA, et al (2011) Quantification of hepatic steatosis with T1-independent, T2-corrected MR imaging with spectral modeling of fat: Blinded comparison with MR spectroscopy. Radiology 258: 767–775. 21248233 10.1148/radiol.10100708 PMC3042638

[46] Bottomley PA, Hardy CJ, Argersinger RE, Allen-Moore G (1987) A review of 1H nuclear magnetic resonance relaxation in pathology: Are T1 and T2 diagnostic? Med Phys 14: 1–37. 3031439 10.1118/1.596111

[47] Yu H, McKenzie CA, Shimakawa A, Vu AT, Brau AC, et al (2007) Multiecho reconstruction for simultaneous water-fat decomposition and T2* estimation. J Magn Reson Imaging 26: 1153–1161. 17896369 10.1002/jmri.21090

[48] Dyvorne HA, Galea N, Nevers T, Fiel MI, Carpenter D, et al (2013) Diffusion-weighted imaging of the liver with multiple b values: Effect of diffusion gradient polarity and breathing acquisition on image quality and intravoxel incoherent motion parameters—a pilot study. Radiology 266: 920–929. 23220895 10.1148/radiol.12120686 PMC3579172

[49] Patel J, Sigmund EE, Rusinek H, Oei M, Babb JS, et al (2010) Diagnosis of cirrhosis with intravoxel incoherent motion diffusion MRI and dynamic contrast-enhanced MRI alone and in combination: Preliminary experience J Magn Reson Imaging. 31: 589–600. 10.1002/jmri.22081 PMC5207803 20187201

[50] Rao RK, Riffel P, Meyer M, Kettnaker PJ, Lemke A, et al (2012) Implementation of dual-source RF excitation in 3 T MR-scanners allows for nearly identical ADC values compared to 1.5 T MR scanners in the abdomen. PLoS One 7: e32613. 22393422 10.1371/journal.pone.0032613 PMC3290586

[51] Liska D, Dufour S, Zern TL, Taksali S, Cali AM, et al (2007) Interethnic differences in muscle, liver and abdominal fat partitioning in obese adolescents. PLoS One 2: e569. 17593968 10.1371/journal.pone.0000569 PMC1892806

[52] Chandarana H, Lim RP, Jensen JH, Hajdu CH, Losada M, et al (2009) Hepatic iron deposition in patients with liver disease: Preliminary experience with breath-hold multiecho T2*-weighted sequence. AJR Am J Roentgenol 193: 1261–1267. 19843739 10.2214/AJR.08.1996

[53] Hiwatashi A, Kinoshita T, Moritani T, Wang HZ, Shrier DA, et al (2003) Hypointensity on diffusion-weighted MRI of the brain related to T2 shortening and susceptibility effects. AJR Am J Roentgenol 181: 1705–1709. 14627600 10.2214/ajr.181.6.1811705

